# Cystic Fibrosis Transmembrane Conductance Regulator Modulators During Pregnancy: A Case Series

**DOI:** 10.7759/cureus.17427

**Published:** 2021-08-25

**Authors:** Anthony M Kendle, Jared T Roekner, Elsa C Santillana, Lilla E Kis, Mary A Cain

**Affiliations:** 1 Obstetrics and Gynecology, University of South Florida, Tampa, USA; 2 Pulmonary and Critical Care Medicine, University of South Florida, Tampa, USA

**Keywords:** cystic fibrosis, pregnancy, transmembrane conductance regulators modulators, medication exposure, contraception

## Abstract

Cystic fibrosis (CF) is the most common genetic disease in the United States (US) and, with the development of newer therapeutics, there is increased fertility among women with CF. We present a series of pregnant patients taking novel CF transmembrane conductance regulator (CFTR) modulators and summarize pertinent clinical considerations. All women conceived within four months after starting elexacaftor-ivacaftor-tezacaftor. Pulmonary function was stable before and during pregnancy. One patient developed transaminitis necessitating discontinuation of the medication mid-trimester. All patients delivered healthy neonates between 36-38 weeks of gestation with uncomplicated postpartum courses. No birth defects were encountered. Given that newly introduced CFTR modulators may increase fertility among CF patients, contraception counseling, pulmonary function monitoring, liver function monitoring, and multi-disciplinary care are important pillars of management.

## Introduction

Cystic fibrosis (CF) results from an autosomal recessive mutation in the CF transmembrane conductance regulator (CFTR) protein causing derangements of sodium and chloride ion transport that result in progressive lung dysfunction and gastrointestinal, exocrine, endocrine, and reproductive alterations [1]. For women with CF, unique considerations surround pre-conception and antepartum management. Subfertility in this population is well established with plausible mechanisms including increased viscosity of cervical mucus, alterations in vaginal pH, anovulation, pancreatic insufficiency, and maternal malnutrition [2]. Nutritional optimization, preconception counseling to discuss risks of preterm birth, low birth weight, cesarean delivery, and multidisciplinary care with close monitoring of respiratory function, weight gain, and fetal well-being are important considerations [3].

In 2019 the FDA approved a triple combination CFTR modulator therapy, elexacaftor-ivacaftor-tezacaftor, for patients with at least one F508del mutation, which affects about 90% of the CF population. While this drug class restores aberrant protein folding and increases expression at the cell membrane resulting in improved outcomes, limited data exist regarding pregnancy among women treated with CFTR modulators [1]. We present a series of five women who became pregnant shortly after initiation of the CFTR modulator combination therapy of elexacaftor-ivacaftor-tezacaftor.

## Case presentation

The first patient is a 29-year-old G1P0 Hispanic female with CF (genotype F508del/R1066C, pre-pregnancy BMI 18.5 kg/m2, baseline forced expiratory volume (FEV1) 94% predicted) who began prenatal care at 12 weeks. Her medical history was significant for CF-associated diabetes, anxiety, chronic gastritis, malnutrition, pancreatic insufficiency, and methicillin-resistant *Staphylococcus aureus* (MRSA) colonization. Surgical history was significant for gastrostomy tube placement (removed prior to pregnancy). She was started on elexacaftor-ivacaftor-tezacaftor four months prior to conception. At approximately six weeks gestation, the elevation of liver enzymes (AST 137 U/L and ALT 218 U/L) was noted on routine monitoring. Additional workup did not suggest alternative etiology to drug side effects. She discontinued elexacaftor-ivacaftor-tezacaftor until normalization of hepatic function and restarted the medication at 31 weeks gestation. Liver function remained within normal ranges by serial testing. Her diabetes was well controlled on insulin. Pulmonary function tests (PFTs) during pregnancy remained stable (FEV1 85% predicted at 34 weeks). She presented at 38w0d gestation with prelabor rupture of membranes, developed chorioamnionitis, and ultimately underwent a cesarean delivery of a female infant, 3020g, with APGARs of 9/9. There were no neonatal complications. The patient was discharged on postoperative day two, breastfed the infant, and received a levonorgestrel intrauterine system for contraception.

The second patient is a 28-year-old G2P1001 Hispanic female with CF (genotype F508del/P205S, pre-pregnancy BMI 33.6 kg/m2, baseline FEV1 56.6% predicted) who began prenatal care at 10 weeks gestation. She delivered a full-term infant by vaginal delivery at age 19, but for the past seven years, she reported an inability to conceive. She never underwent an infertility workup. Past medical history was significant for asthma, gastroesophageal reflux, chronic sinusitis, and MRSA colonization. Surgical history was significant for multiple sinus surgeries. She unintentionally conceived two months after starting elexacaftor-ivacaftor-tezacaftor. PFTs during pregnancy remained stable (FEV1 63% predicted at 35 weeks). Due to pruritis and elevated bile acids, she was diagnosed with intrahepatic cholestasis of pregnancy at 34 weeks and underwent induction of labor at 36w4d. She vaginally delivered a male infant weighing 2790g with APGARs 9/9. There were no maternal or neonatal complications. She was discharged on postpartum day two, breast- and bottle-fed the baby, and received an etonogestrel contraceptive implant.

Patient 3 is a 28-year-old G1P0 Caucasian female with CF (genotype homozygous F508del, pre-pregnancy BMI 21.5 kg/m2, baseline FEV1 65% predicted) who began prenatal care at eight weeks. Her medical history was significant for malnutrition, pancreatic insufficiency, chronic sinusitis, anxiety and depression, gastroesophageal reflux, endometriosis, MRSA colonization, and pseudomonas colonization. Her surgical history included bronchial embolization and sinus surgeries. She started elexacaftor-ivacaftor-tezacaftor one month prior to conception. Pregnancy PFTs remained stable (FEV1 70% predicted at 34 weeks). She had a spontaneous vaginal delivery at 38w4d of a female infant, 2980g with APGARs of 9/9. There were no maternal or neonatal complications. She was discharged on postpartum day two, breastfed, and declined any form of contraception other than barrier methods.

The fourth patient is a 29-year-old G2P0010 Caucasian female with CF (genotype F508del/G551D, pre-pregnancy BMI 16.7 kg/m2, baseline FEV1 66%) who began prenatal care at 13 weeks. Her medical history was significant for allergic rhinitis, chronic sinusitis, constipation, gastroesophageal reflux, pancreatic insufficiency, pancreatic cyst, kidney stone, anxiety, depression, *Burkholderia cepacia* colonization, and MRSA colonization. She started elexacaftor-ivacaftor-tezacaftor within the same month as conception. Pregnancy PFTs were stable (FEV1 61% at 31 weeks). She underwent induction of labor at 39wks for fetal growth restriction and delivered a female infant, 2760g, with APGARs of 9/9. There were no maternal or neonatal complications, and she was discharged on postpartum day two, breast- and bottle-fed the infant. She was undecided on contraception at the time of discharge.

The fifth patient is a 23-year-old G4P0030 Hispanic female with CF (genotype F508del/C3791del, pre-pregnancy BMI 26 kg/m2, baseline FEV1 36.5%). Past medical history was significant for polycystic ovary syndrome (PCOS), fibromyalgia, scoliosis, arthritis, pancreatic insufficiency, and chronic maxillary sinusitis. Surgical history included sinus surgery, tonsillectomy, tracheostomy, multiple colon surgeries, colostomy with reversal, ileostomy with reversal, Nissen fundoplication, and gastrostomy tube placement. Prior pregnancies resulted in spontaneous abortion. She started elexacaftor-ivacaftor-tezacaftor one month prior to conception. She had two CF exacerbations during pregnancy. The first occurred in early pregnancy prior to transfer to our institution and required a two-week hospital stay. The second exacerbation occurred at 31w5d. Sputum culture showed *Pseudomonas aeruginosa*, and she received one week of IV vancomycin and cefepime. Pregnancy FEV1 increased to 54.4% predicated at 35 weeks. She had a spontaneous vaginal delivery at 37w0d and delivered a female infant, 2780g, with APGARs of 8/9. There were no neonatal complications. On postpartum day one, she developed transient transaminitis (AST 74 U/L, ALT 60 U/L). Liver enzymes decreased (AST 51 U/L, ALT 40 U/L) on postpartum day two, and the pulmonologist recommended the continuation of CFTR modulator therapy. She was discharged on postpartum day two, bottle-fed the infant, and received oral norethindrone for contraception.

A summary of patient characteristics and pregnancy outcomes is displayed in Table 1.

**Table 1:** Comparison of patient characteristics and pregnancy outcomes CF: cystic fibrosis; FEV1: forced expiratory volume in 1 second; GA, gestational age

	Patient 1	Patient 2	Patient 3	Patient 4	Patient 5
Age	29	28	28	29	23
CF genotype	F508del/R1066C	F508del/P205S	F508del/F508del	F508del/G441D	F508del/C3791del
Pregravid BMI (kg/m^2^)	18.5	33.6	21.5	16.7	26.0
Total weight gain (kg)	6.6	19.1	11.8	12	8.5
Baseline FEV1 (% predicted)	94	56.6	65	66	36.5
FEV1 prior to delivery (% predicted)	85	63	70	61	54.4
GA at delivery	38w0d	36w4d	38w4d	39w1d	37w0d
Delivery route	cesarean	vaginal	vaginal	vaginal	vaginal
Infant birth weight (g)	3020	2790	2980	2760	2780
Antenatal complications	Elevation of serum transaminases	Intraheptaic cholestasis	None	None	Two antenatal CF exacerbations

## Discussion

All pregnancies in our case series were unplanned, occurring within four months of starting treatment, highlighting the importance of contraception in the era of CFTR modulator therapy. Only 35% of women with CF use contraception, and low utilization rates, misconceptions regarding fertility, and potential metabolic alterations of hormonal contraception contribute to a high rate of unplanned pregnancy (26-50%) [3]. According to the Cystic Fibrosis Foundation Patient Registry, pregnancy rates increased from 14.4/1000 women-years to 38.4/1000 after approval of ivacaftor in 2012 [4], and the effect of CFTR modulator therapy on fertility may manifest rapidly. 

Drug exposure counseling and medication reconciliation are important for the maternal-fetal dyad. Safety data for CFTR modulator therapy is limited and CYP3A4/5 metabolism may cause drug-drug interactions. While teratogenesis was not seen in rat studies, CFTR modulator levels were detected in rat placentas and breast milk [5]. Although drug transfer occurs through the human placenta and breast milk, reports demonstrate favorable pregnancy outcomes. One study examining aspartate aminotransferase and bilirubin in breastfed infants noted that elevations were not distinguishable from a physiologic variation of lab values in neonatal life [6]. Mothers using CFTR modulators who choose to breastfeed should inform their pediatrician to ensure appropriate infant surveillance.

The first case in our series developed transaminitis suspected secondary to treatment with elexacaftor-ivacaftor-tezacaftor prompting drug discontinuation. The fifth case in our series also developed mild transient transaminitis postpartum. Of the patients taking elexacaftor-ivacaftor-tezacaftor, 5-10% demonstrate elevations in transaminases and bilirubin; therefore it is recommended to obtain levels prior to initiation, every three months after commencement for the first year, and annually thereafter [7]. Although recommendations for monitoring after drug initiation are established, there is no clear guidance regarding frequency of monitoring during pregnancy, though periodic liver function assessment is reasonable. Decisions regarding discontinuation and re-initiation of elexacaftor-ivacaftor-tezacaftor during pregnancy should be made in conjunction with the prescribing pulmonologist and perinatologist, and other etiologies for transaminitis such as preeclampsia or intrahepatic cholestasis should remain in the differential.

Appropriate nutrition is key for fetal growth. While many pregnant women with CF struggle to reach recommended weight gain goals, weight gain in this series was appropriate (mean 11.6 ± 4.7 kg). Pulmonary complications have also been seen in CF mothers with FEV1 < 60%. Two patients from this series had a pre-pregnancy FEV1 below this threshold, and all patients except for the fifth case had an FEV1 above 60% prior to delivery. The fifth case had a baseline FEV1 of 36.5% and an FEV1 of 54.4% in the third trimester and was the only patient in this series to require hospitalizations for pulmonary exacerbation during her pregnancy.

## Conclusions

The future of CF care has changed dramatically in the past decade with the advent of CFTR modulator therapy. Early initiation holds promise to prevent irreversible pulmonary and exocrine function. As a result, we will continue to see an increase in pregnant women using CFTR modulators. Clinicians should understand the effects of these medications on fertility and counsel patients on appropriate contraception and pregnancy risks. Although the pregnancy outcomes were positive among these five patients taking elexacaftor-ivacaftor-tezacaftor, future research with larger cohorts is necessary to assess pregnancy outcomes and drug safety.
